# Editorial European Journal of Nuclear Medicine and Molecular Imaging

**DOI:** 10.1007/s00259-016-3559-2

**Published:** 2016-11-07

**Authors:** Ambros J. Beer, Ingrid Dijkgraaf

**Affiliations:** 1 Department of Nuclear Medicine, Ulm University, Ulm, Germany; 2 Department of Biochemistry, Maastricht University, P.O. Box 6200 MD, Maastricht, The Netherlands

In this issue of The European Journal of Nuclear Medicine and Molecular Imaging, Xiaohui Luan et al. at Shandong Cancer Hospital report the role of ^18^F-alfatide positron emission tomography/computed tomography (PET/CT) for assessment of integrin αvβ3 expression in predicting the short-term outcome of concurrent chemoradiotherapy (CCRT) in patients with advanced non-small cell lung cancer (NSCLC). There is a need for an effective predicting tool to select patients with advanced NSCLC who may benefit from CCRT, which represents the standard of therapy protocol, because one-third of these patients experience local failure. Molecular imaging of specific biological targets known to play a role in tumour biology and aggressiveness lends itself for this purpose. Especially, the integrin αvβ3 has gained interest in this respect as it is known to be overexpressed on activated endothelial cells in angiogenesis and also on many tumour cells and plays an important role in cell–cell and cell–matrix interactions in general. In the past two decades, the potential of using radio-labelled arginine-glycine-aspartic acid (RGD)-containing﻿ peptides to serve as imaging agents for noninvasive evaluation of integrin expression has been profoundly investigated by several research groups. For the development of a clinically suitable αvβ3 integrin-binding radiopharmaceutical, and radiopharmaceuticals in general, several challenges have to be taken into account. Aside from sublime in vivo stability, high receptor affinity, favorable pharmacokinetics and pharmacodynamics, and low toxicity and immunogenicity, the radiolabelling procedure should be rapid and without the necessity of laborious purification steps.

The first clinically applied RGD-based PET radiotracer which meets most of the above-mentioned conditions for imaging αvβ3 expression in tumours was [^18^F]Galacto-RGD. This radiotracer cleared rapidly from the blood pool, showed predominantly renal excretion, and could successfully image integrin expression in human tumours with good tumour-to-background ratios [1–3]. It appeared that molecular imaging of αvβ3 expression, with [^18^F]Galacto-RGD in humans correlated with αvβ3 expression, as determined by immunohistochemistry [4].

Another ^18^F-labelled RGD peptide that has clinically been tested is ^18^F-AH111585 [5]. This PET tracer was demonstrated to be safe and well tolerated, with no adverse events in the patients studied. All primary and metastatic lesions that were identified on CT within the PET field of view were detected with ^18^F-AH111585 PET.

However, the PET radiotracers described above are peptides based on monomeric cyclic RGD and comparison studies of integrin-binding affinities of multimeric (i.e. dimeric, tetrameric, and octameric) RGD peptides with their monomeric analogs revealed significantly increasing affinities in the series monomer < dimer < tetramer < octamer [6–10] . In general, both tumour uptake and tumour-to-organ ratios increased for multimeric RGD peptides, resulting in improved tumour imaging. However, it is important to note that RGD-multimerisation has its limitations as a higher degree of oligomerisation not only led to a higher tumour uptake of the tracer, but also caused a higher tracer uptake in non-target tissues, especially the kidneys. This will decrease detection sensitivity in the proximity of the kidneys and could cause radiation nephrotoxicity in therapeutic applications which necessitates use of other tracer optimisation strategies.

Besides the fact that [^18^F]Galacto-RGD and ^18^F-AH111585 are monomeric RGD-based agents, both tracers are radiofluorinated via prosthetic groups which involves multistep time-consuming and low-yield synthetic procedures, limiting their widespread use as routine tracers in the clinic. The recent development of [^18^F]-fluoride–aluminum complexes to radiolabel peptides via chelation chemistry simplified and shortened radiofluorination of peptides and even allows kit formulation, facilitating clinical translation [11].

The newly developed ^18^F-AlF-NOTA-PRGD_2_, denoted as ^18^F-alfatide, combines both utilities: facile one-pot Al^18^F-radiofluorination and multivalency by having two RGD moieties [12, 13]. After optimisation of radiolabelling conditions (e.g. temperature and pH), the whole radiosynthesis and subsequent purification can be performed within 20 min with a decay-corrected yield of 42.1 ± 2.0 % and radiochemical purity of more than 95 %. In the first clinical studies, ^18^F-alfatide-PET allowed specific imaging of αvβ3 expression in lung cancers with good contrast [14]. Additionally, it appeared that ^18^F-alfatide-PET/CT parameters can predict tumor sensitivity to CCRT in patients with glioma [15]. From the ^18^F-alfatide-PET/CT study, described in this European Journal of Nuclear Medicine and Molecular Imaging’s issue, it appeared that the maximum standardized uptake value (SUV_max_) , SUV_peak_, T/NT_lung_, T/NT_blood_, and T/NT_muscle_ were higher in non-responders than responders and that ^18^F-alfatide PET/CT might be useful in predicting the short-term outcome of CCRT in patients with advanced NSCLC.

While this is an interesting preliminary study on the potential predictive value of ^18^F-alfatide PET/CT in patients with NSCLC before undergoing CRRT, final conclusions cannot yet be drawn on the ultimate clinical usefulness of this approach in the presented setting. Now, studies with larger patient collectives are warranted to corroborate the presented results. Moreover, a comparison to other functional and molecular imaging techniques for evaluation of tumour biology, patient prognosis and response to therapy should be performed as well, like FDG PET/CT or perfusion/diffusion imaging with magnetic resonance imging (MRI).
